# Using Kano diagrams to display the most cited article types, affiliated countries, authors and MeSH terms on spinal surgery in recent 12 years

**DOI:** 10.1186/s40001-021-00494-x

**Published:** 2021-02-23

**Authors:** Po-Hsin Chou, Yu-Tsen Yeh, Wei-Chih Kan, Tsair-Wei Chien, Shu-Chun Kuo

**Affiliations:** 1 Department of Orthopedics and Traumatology, Taipei Veterans General Hospital, Taipei, Taiwan; 2 School of Medicine, National Yang-Ming University, Taipei, Taiwan; 3 Medical School, St. George’s, University of London, London, UK; 4 Department of Nephrology, Chi-Mei Medical Center, Tainan, Taiwan; 5 Department of Medical Research, Chi-Mei Medical Center, Tainan, Taiwan; 6 Department of Optometry, Chung Hwa University of Medical Technology, Jen-Teh, Tainan, Taiwan; 7 Department of Ophthalmology, Chi-Mei Medical Center, 901 Chung Hwa Road, Yung Kung, Yong Kang, Tainan, Taiwan

**Keywords:** Spine, Research productivity, Citation analysis, Kano diagram, Choropleth map, PubMed central

## Abstract

**Background:**

Citation analysis has been increasingly applied to assess the quantity and quality of scientific research in various fields worldwide. However, these analyses on spinal surgery do not provide visualization of results. This study aims (1) to evaluate the worldwide research citations and publications on spinal surgery and (2) to provide visual representations using Kano diagrams onto the research analysis for spinal surgeons and researchers.

**Methods:**

Article abstracts published between 2007 and 2018 were downloaded from PubMed Central (PMC) in 5 journals, including *Spine, European Spine Journal, The Spine Journal, Journal of Neurosurgery: Spine, and Journal of Spinal Disorders and Techniques*. The article types, affiliated countries, authors, and Medical subject headings (MeSH terms) were analyzed by the number of article citations using x-index. Choropleth maps and Kano diagrams were applied to present these results. The trends of MeSH terms over the years were plotted and analyzed.

**Results:**

A total of 18,808 publications were extracted from the PMC database, and 17,245 were affiliated to countries/areas. The 12-year impact factor for the five spine journals is 5.758. We observed that (1) the largest number of articles on spinal surgery was from North America (6417, 37.21%). *Spine* earns the highest x-index (= 82.96). Comparative Study has the highest x-index (= 66.74) among all article types. (2) The United States performed exceptionally in x-indexes (= 56.86 and 44.5) on both analyses done on the total 18,808 and the top 100 most cited articles, respectively. The most influential author whose x-index reaches 15.11 was Simon Dagenais from the US. (3) The most cited MeSH term with an x-index of 23.05 was surgery based on the top 100 most cited articles. The most cited article (PMID = 18164449) was written by Dagenais and his colleagues in 2008. The most productive author was Michael G. Fehlings, whose x-index and the author's impact factor are 13.57(= √(13.16*14)) and 9.86(= 331.57/33.64), respectively.

**Conclusions:**

There was a rapidly increasing scientific productivity in the field of spinal surgery in the past 12 years. The US has extraordinary contributions to the publications. Furthermore, China and Japan have increasing numbers of publications on spinal surgery. This study with Kano diagrams provides an insight into the research for spinal surgeons and researchers.

## Background

The number of publications on spinal surgery has dramatically increased in recent years [1–3]. The citation analysis has been applied to measure individual research achievements (IRA) [4–9]. However, several changes have been encountered in those studies using bibliometric analyses, such as all factors (e.g., authors or affiliated countries/areas) in an article are viewed with equal contribution to an article. As such, the research achievements might be unfair and biased in IRA measurement. Two major difficulties we encountered in our study include the following: (1) coauthors with equal credits in an article byline are unreasonable; and (2) the Hirsch’s h-index [7] has less discrimination power due to its integer nature (i.e., many of the same index value) making it difficult to differentiate the personal IRA [10].

The total citation count (e.g., author impact factor, AIF = citations/publications for each author) [4, 5]) has the disadvantage of favoring researchers with few highly cited top publications or productive authors with many publications, but a relatively small number of citations [4, 5].

While the *h*-index is determined by the maximum square that fits under the citation curve of an author when plotting the number of citations in decreasing order, the *x*-index [= $\sqrt {\mathop {\max }\limits_{i} (i \times c_{i} )}$, where all the number of citations (denoted by *ci*) in the *x* core at publication [9] was proposed in 2018 and determined by the maximum rectangular area that fits under the curve. The disadvantage of the *x*-index is the equal importance placed on the citations and publications, regardless of the IRA tendency toward the citations or the publications. The Kano diagram [11] is thus suitable for interpreting the feature for factors in three parts: the excitement, the performance, and the basic requirement denoting the IRAs as the influential, the prolific, and the productive, respectively, in the previous articles [12, 13].

Although many studies [14–21] have used bibliometric methods for evaluating worldwide research productivity in many biomedical fields, including those published in general orthopedics and its subspecialties [22–24], bibliometric analyses on the quantity and quality of articles published in spine journals worldwide are still rare [2]. Only three [1–3] were found searching keyword (spine surgery [Title]) AND bibliometric [MeSH Major Topic] on PubMed Central (PMC) as of November 11, 2019, but all of them lack substantial visualizations in results.

Therefore, the purpose of this study was (1) to evaluate the research citations and publications in major spine journals using the PMC database in recent 12 years and (2) to provide visual representations using the visual Kano diagrams onto the spinal research to display IRAs for surgeons and researchers.

## Materials and methods

### Data source

We searched the PMC using “(((((((((1529–9430) OR 0940–6719) OR 1432–0932) OR 0362–2436) OR 1528–1159) OR 1547–5654) OR 1547–5646) OR 1536–0652) OR 1539–2465) AND ("2007"[Date—Publication]: "2018"[Date—Publication])” on November 2, 2019, for retrieving publications in five relevant journals, including *Spine, European Spine Journal, The Spine Journal, Journal of Neurosurgery: Spine, and Journal of Spinal Disorders and Techniques*. In total, we downloaded 18,808 abstracts that were published from 2007 to 2018, see Additional file 1.

All data used in this study were downloaded from the PMC, meaning that the study did not require ethical approval according to the regulations promulgated by the Taiwan Ministry of Health and Welfare.

## Two prerequisites used for evaluating author IRAs

An author-weighted scheme (AWS) was applied to determine coauthors’ contributions in article bylines [25] to improve the unreasonable phenomenon that all papers are equal weight irrespective of the number of coauthors [26, 27]. Accordingly, more importance was given to the first (primary) authors and the last (corresponding/supervisory) authors, while we assumed that the others (i.e., the middle authors) made smaller contributions [25].

Furthermore, the *x*-index [9] used in this study generalizes the *h*-index [7], which is determined by the square area (i.e., the number of publications equal to the citation point). The *x*-index thus surpasses the h-index in differentiating the personal IRA among affiliated countries/areas, authors, or the medical subject headings (MeSH terms) [28].

## Task 1: *x*-index for journals and article types

The trend of the number of articles across continents worldwide and journals over time was shown in a contingency table based on the 1st authors affiliated to their countries/areas. Journals and article types were compared to identify the most influential ones with either a higher *x*-index or *h*-index.

## Task 2: *x*-index for affiliated countries/areas and authors

Three plots of the choropleth map [29], the bar chart, and the Kano model [11] were applied to display IRA features for affiliated countries/areas and authors based on the 18,808 abstracts and the top 100 most cited articles, respectively.

## Task 3: *x*-index for MeSH terms

MeSH terms extracted from the top 100 most cited articles were represented by the topic burst on the topic of spine surgery [30]. Three plots of the Kano diagram, the line chart, and the clusters using social network analysis(SNA) [31] were used for interpreting (1) the evolution of topics in the past 12 years, (2) the most cited terms, and (3) the MeSH clusters are separated by the SNA using Pajek software [32].

The most cited article and the most productive authors in these 18,808 abstracts were extracted separately from the analysis performed on an author-made routine with codes of visual basic for application(VBA) on Microsoft Excel.

## Results

### *x*-Index for journals and article types

A total of 18,808 publications were extracted, of those 17,245 were identified with affiliated countries/areas, and 46,795 authors were included. The 12-year impact factor for the five journals together is 5.758. The largest number of articles on spinal surgery was from North America (6417, 37.21%); see Table 1. The journal *Spine* earns the highest *x*-index (= 82.96); see Table 2. Comparative study has the highest *x*-index (= 66.74) among article types; see Table 3.

**Table 1:** Publications across continents and journals over the years based on 1st authors

Continent/journal	2007	2008	2009	2010	2011	2012	2013	2014	2015	2016	2017	2018	Total	%
AFRICA	2	4	3	3	12	10	9	8	5	17	3	8	84	0.49
Eur Spine J		1	1	3	2	4	4	2	1	12		1	31	0.18
J Neurosurg Spine					3				1		1	2	7	0.04
J Spinal Disord Tech					1								1	0.01
Spine (Phila Pa 1976)	2	3	1		2		4	2			2	4	20	0.12
Spine J			1		4	6	1	4	3	5		1	25	0.14
ASIA	345	339	379	406	450	439	533	545	658	731	465	522	5812	33.70
Eur Spine J	64	77	64	79	122	121	163	146	187	221	187	203	1634	9.48
J Neurosurg Spine	73	56	50	66	54	40	53	33	43	40	33	32	573	3.32
J Spinal Disord Tech	39	38	38	40	46	55	64	58	54				432	2.51
Spine (Phila Pa 1976)	146	146	194	191	193	192	167	181	149	191	172	178	2100	12.18
Spine J	23	22	33	30	35	31	86	127	225	279	73	109	1073	6.22
EUROPE	349	306	319	333	354	381	428	390	374	368	321	375	4298	24.92
Eur Spine J	148	110	151	149	177	229	239	199	188	217	183	221	2211	12.82
J Neurosurg Spine	37	29	21	27	18	16	11	8	13	22	19	13	234	1.36
J Spinal Disord Tech	13	13	8	17	17	15	12	9	15				119	0.69
Spine (Phila Pa 1976)	140	142	123	127	109	95	113	81	75	65	69	61	1200	6.96
Spine J	11	12	16	13	33	26	53	93	83	64	50	80	534	3.10
N. AMERICA	538	533	560	583	552	579	581	519	520	518	413	521	6417	37.21
Eur Spine J	44	25	33	34	23	51	48	36	69	79	56	93	591	3.43
J Neurosurg Spine	89	81	108	96	119	124	97	71	76	107	79	84	1131	6.56
J Spinal Disord Tech	48	51	51	41	35	39	37	24	39				365	2.12
Spine (Phila Pa 1976)	285	282	255	295	281	256	245	182	147	177	174	149	2728	15.82
Spine J	72	94	113	117	94	109	154	206	189	155	104	195	1602	9.29
OCEANIA	40	38	37	37	44	30	26	24	48	39	39	55	457	2.65
Eur Spine J	11	7	13	10	17	11	6	7	12	16	14	18	142	0.82
J Neurosurg Spine	1	4	3	5	2	4	2		2	1		2	26	0.15
J Spinal Disord Tech	3	2	1		2	1	2	1					12	0.07
Spine (Phila Pa 1976)	23	22	19	19	21	9	13	9	14	13	14	20	196	1.14
Spine J	2	3	1	3	2	5	3	7	20	9	11	15	81	0.47
S. AMERICA	9	13	13	8	19	11	14	14	15	26	10	24	176	1.02
Eur Spine J	1	3	1	3	5	6	4	4	4	8	3	10	52	0.30
J Neurosurg Spine	4	3	2		4	1	2	2		2	3		23	0.13
J Spinal Disord Tech					2		1	1	1				5	0.03
Spine (Phila Pa 1976)	3	6	8	4	6	3	4	4	5	6	4	9	62	0.36
Spine J	1	1	2	1	2	1	3	3	5	10		5	34	0.20
Total	1283	1233	1311	1370	1431	1450	1591	1500	1621	1699	1251	1505	17,245	100.00

**Table 2:** Comparison of SCI IF and PubMed IF for journals

Journal	SCI IF (2 years)	Citable	cited	JIF (10 years)	*h*	*Ci*	*k*	*x*
Spine (Phila Pa 1976)	2.903	6941	49,677	7.157	33	6	1147	82.96
Eur Spine J	2.513	4792	25,841	5.393	34	6	1111	81.65
Spine J	3.196	3440	14,998	4.360	37	5	960	69.28
J Neurosurg Spine	2.998	2601	12,576	4.835	32	6	753	67.22
J Spinal Disord Tech	2.310	1034	5202	5.031	24	4	504	44.90
Sum		18,808	108,294	5.758				

The order of publications is similar to the previous study

**Table 3:** Bibliometrics on article types

No.	Article type	*h*	*Ci*	*k*	*x*
1	Comparative Study	33	5.09	875	66.74
2	Case Reports	19	2.19	944	45.50
3	Journal Article	12	2.00	1034	45.43
4	Clinical Trial	18	6.44	131	29.04
5	Review	17	2.94	263	27.79
6	Systematic Review	13	2.84	230	25.57
7	Evaluation Studies	13	4.36	130	23.80
8	Randomized Controlled Trial	9	2.42	169	20.23
9	Multicenter Study	7	1.08	226	15.59
10	Validation Studies	7	1.88	120	15.03

## *x*-Index for affiliated countries/areas and authors

**Fig. 1:**
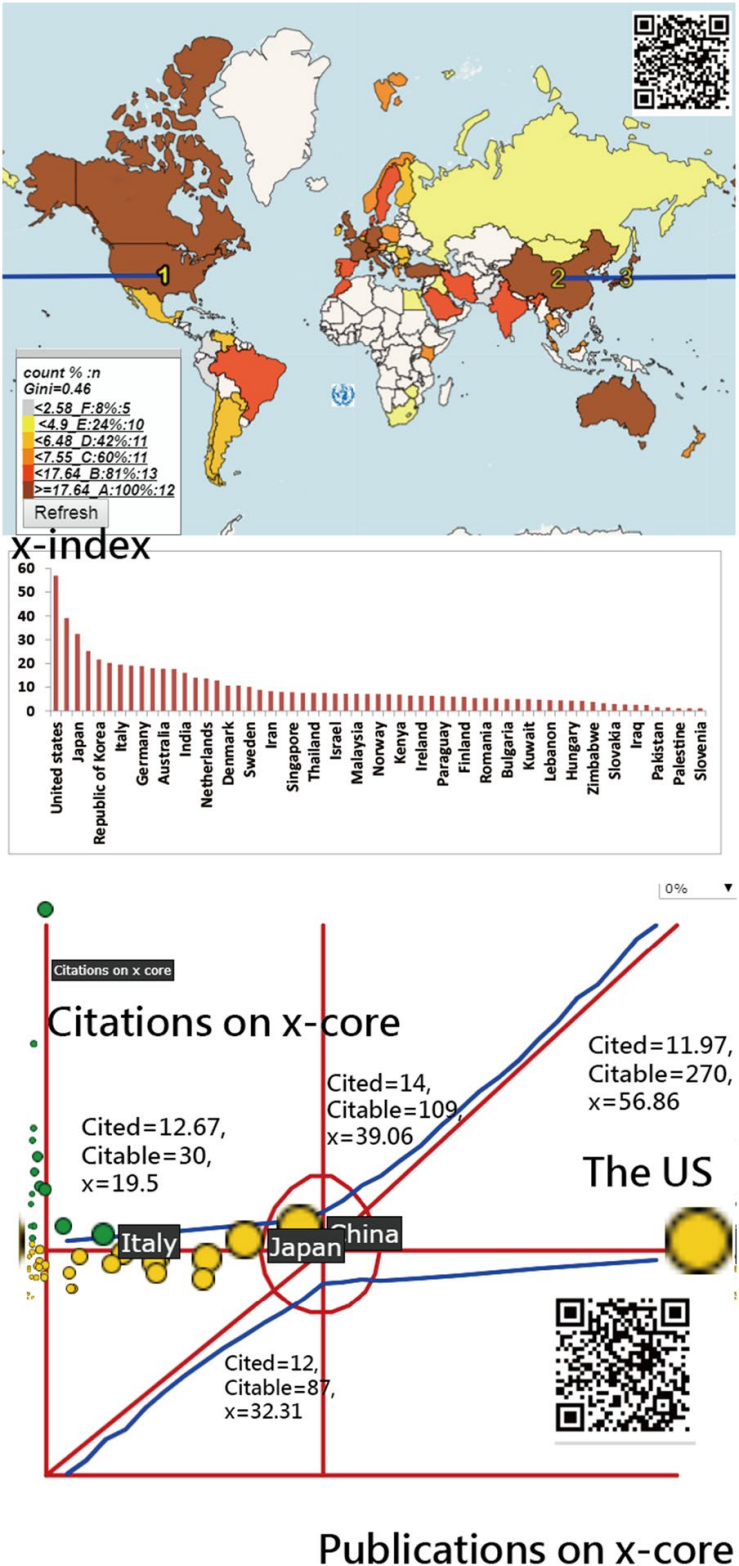
Research achievements for countries on spine using *x*-index and 18,808 publications

**Fig. 2:**
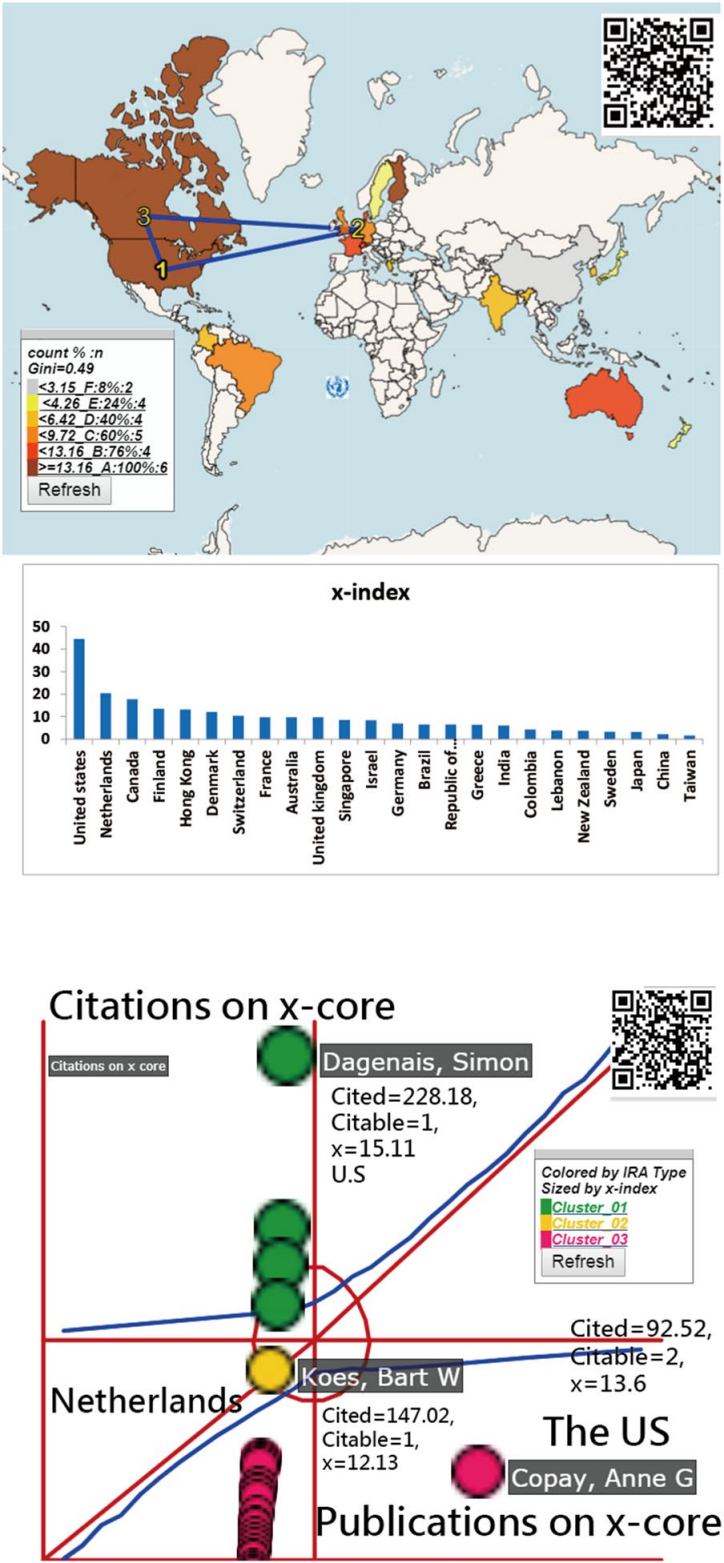
Research achievements for authors on spine using *x*-index and top 100 cited articles

The United States has the highest *x*-indexes (= 56.86 and 44.5) among the world based on the total 18,808 publications and the top 100 most cited articles, respectively; see Fig. 1. The most influential author with the *x*-index = 15.11 is Simon Dagenais from the US; see Fig. 2. We can see the difference in choropleth maps in Figs. 1 and 2, indicating only four countries: the US, Netherlands, Canada, and Finland play an influential role on the topic based on the top 100 most cited articles. Interested readers are invited to scan the QR-code and click the country of interest to view the *x*-index and other details on the information of the entity.

## *x*-Index for MeSH terms

**Fig. 3:**
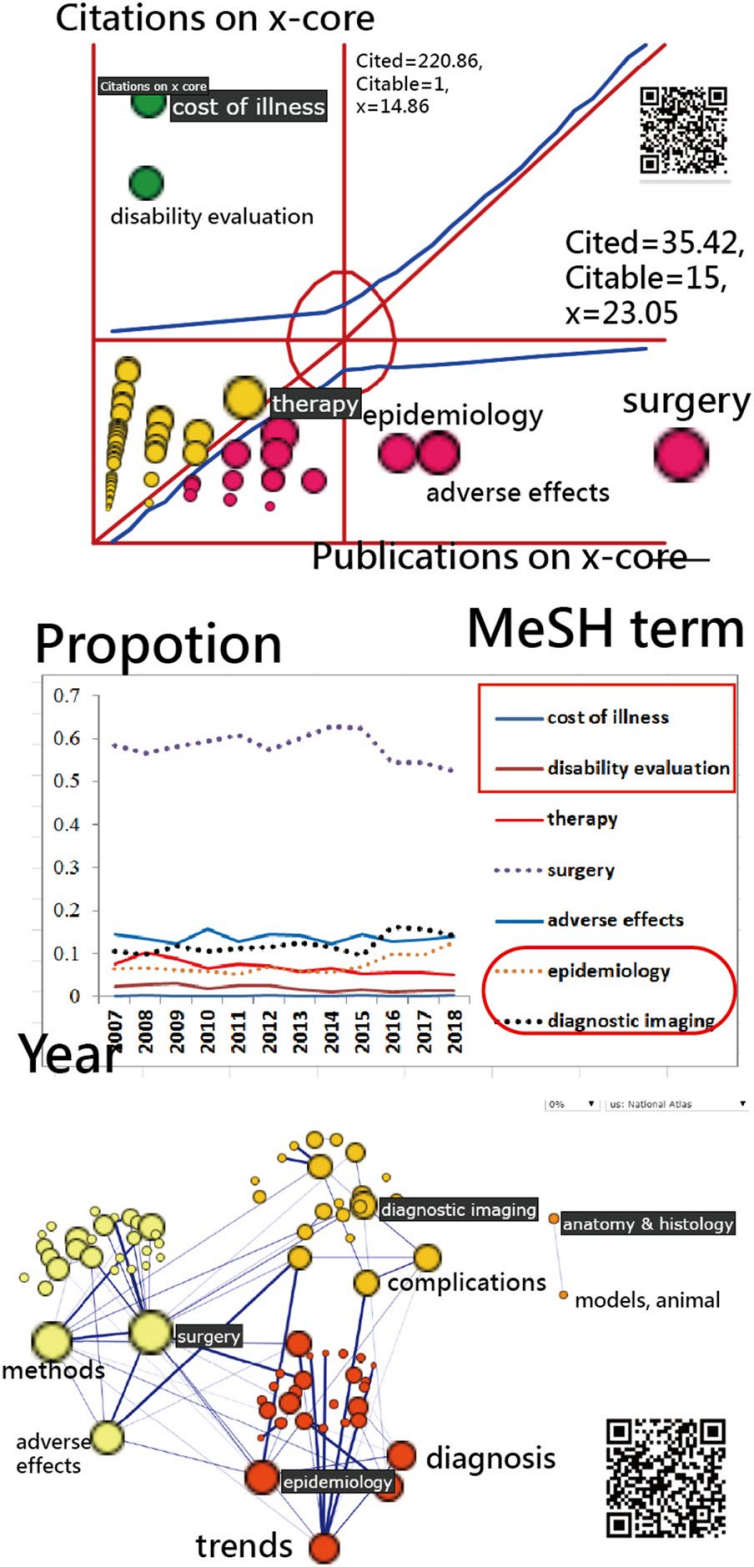
Metrics for the top MeSH terms on spine across the years using *x*-index to display

A total of 82 MeSH terms were retrieved from the top 100 most cited articles. The most cited MeSH term was surgery with the *x*-index = 23.05. We can see the term of surgery with a red bubble on the right-bottom corner in the Kano diagram (top in Fig. 3), indicating the tendency toward its productive feature (i.e., with more weights on publications according to the definition of *x*-index).

The principal MeSH terms extracted from the top panel in Fig. 3 are present at the middle panel of Fig. 3, which shows that both costs of illness and disability evaluation occupy a lower proportion over the years. Both epidemiology and diagnostic imaging have accounted for more since 2016, implying the importance of these topics in spinal surgery in recent years. In contrast, the MeSH term of surgery has been dramatically decreasing since 2016, indicating a relatively smaller number of publications was on the topic of surgery.

The most cited article (PMID = 18,164,449) was written by Dagenais and his colleagues in 2008 [33]. The most productive author is Michael G. Fehlings, with an *x*-index = 13.57 (= √(13.16*14)) and the AIF = 9.86 (= 331.57/33.64 = the cited/the citable computed by the AWS mentioned in Methods 2,2). Interested readers are invited to search the term of (Fehlings, Michael G. [Author—Full]) AND surgery[MeSH Major Topic] to view his publications on PMC.

## Discussion

We observed that the largest number of articles on spinal surgery was from North America (6417, 37.21%) and Asia (5812, 33.7%). The results are different from the findings that the US and Europe are more dominant in science [34, 35].

In the previous study [2], the largest number of publications in the field of spinal surgery was from the US (5137; 39.17%), followed by Japan (1408; 10.74%), and China (1131; 8.62%). The US had the highest h-index (106), followed by Canada (60), and the United Kingdom (54). However, our study observed a different result where the most productive countries are the US (5745, 32.95%), followed by China (1770,10.15%), and Japan (1712, 9.82%). The US also had the highest *x*-index (56.86), followed by China (39.06), and Japan (32.31); see the bottom panel in Fig. 1.

Furthermore, *Spine* earns the highest *x*-index(= 82.96) of all journals, and comparative study has an *x*-index (= 66.74) highest among article types. We have not seen any publication that applied *x*-index in bibliometric analyses to measure IRAs on spinal surgery in literature [1–3, 14–24]. The *x*-index [9] generalizes the *h*-index [7], which is determined by the square area. The drawback of the *x*-index is the equal importance placed on the citations and publications that can be resolved by using the Kano diagram; see Figs. 1, 2, 3, which is a highlight of this study.

The second feature of this study is the AWS used for quantifying the contributions of authors (or their affiliated countries) in an article byline. A fair IRAs could not be achieved; otherwise, see Figs. 1 and 2. Similarly, the weights of MeSH terms can be quantified by the AWS with an equal size (e.g., *Wi* = citations/*n*, where *n* = the number of MeSH terms in an article and *Wi* is the weight of a MeSH term(*i*)) in an article and then plotted in Fig. 3.

Another feature is about the choropleth map incorporated in the bar chart and the Kano diagram as map legends designed for visualizing the metric disparities for better interpretation of values on the choropleth map [36]. For instance, the US with a yellow bubble is the most influential country in the performance area on the bottom panel in Fig. 1, providing a complemental interpretation of the choropleth map at the top panel in Fig. 1. All figures provide QR-codes for interested readers to examine the details on the information with the dashboard on Google Maps, different from those regarding spine-related studies [37, 38] using the world map only available in color online.

We present the most cited MeSH term of surgery with the *x*-index = 23.05 based on the top 100 most cited articles. The method computing IRA for MeSH terms has never been seen before in academics although the placement scheme is similar to the coauthors suitable for quantifying their credits with the AWS in an article.

There are several limitations to this study. First, the five main journals relevant to spinal surgery in this study cannot represent other specialties and the many more basic research journals publishing articles related to spinal surgery. Nevertheless, these five major journals used in this study can represent the study and research contributed to the field.

Second, there might be some biases in author identification because some authors in the bibliometric database may have the same name or use the same abbreviations, but are affiliated to different institutions.

Third, the data extracted from the PMC cannot be generalized to other major citation databases such as the Scientific Citation Index (SCI; Thomson Reuters, New York, NY, the United States) and Scopus (Elsevier, Amsterdam, the Netherlands).

**Fig. 4:**
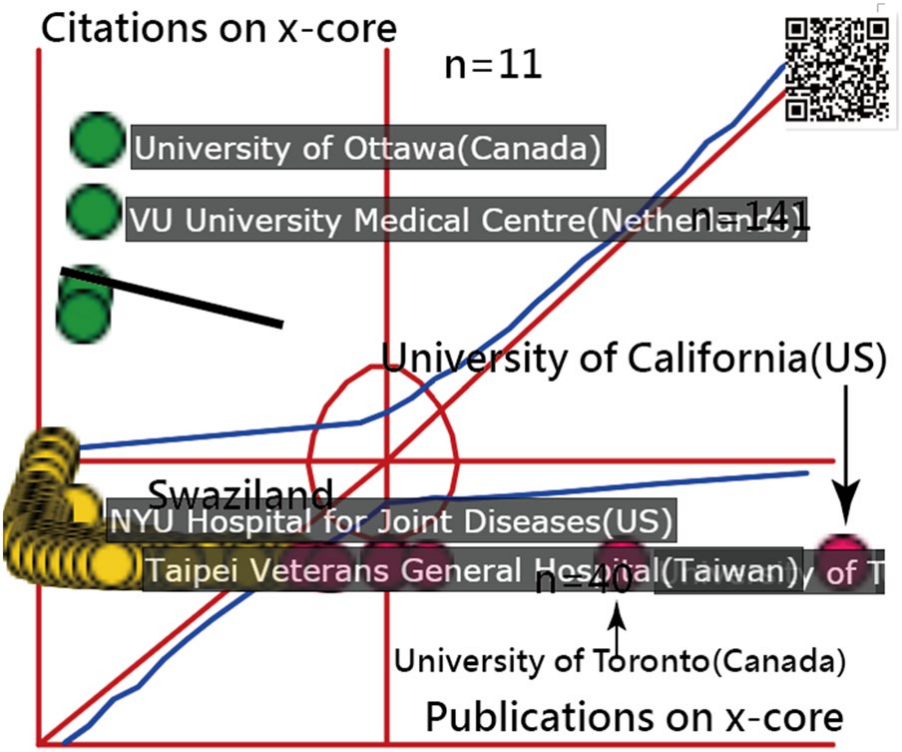
The most cited research institutes using *x*-index to display

Lastly, many researchers believe that the impact factor or citation does not reflect the scientific quality of the research [39–41]. Many journals are now preferring citation distribution curve over the JIF [39]. It is worth discussing how this method for author and journal analyses can be included. Whether the *x*-index[9] that is applied in this study can be used for defining research achievements in a wider range of disciplines is worthy of further studies and discussions in the future (Fig. 4).

## Conclusions

There was a rapid increase in scientific research productivity in the field of spinal surgery in the past 12 years. The US has exceptional contributions to publications. China and Japan have increasing contributions to the field. This study with the visualization by the Kano diagrams provides an insight into the value of research for spinal surgeons and researchers.

## Supplementary Information

**Additional file** 1: Dataset used in this study.

## Data Availability

All data used in this study are available in Additional file files.

## Abbreviations

AWS: Author-weighted scheme
MeSH: Medical subject headings
PMC: Pubmed Center
SCI: Scientific Citation Index
SNA: Social network analysis
